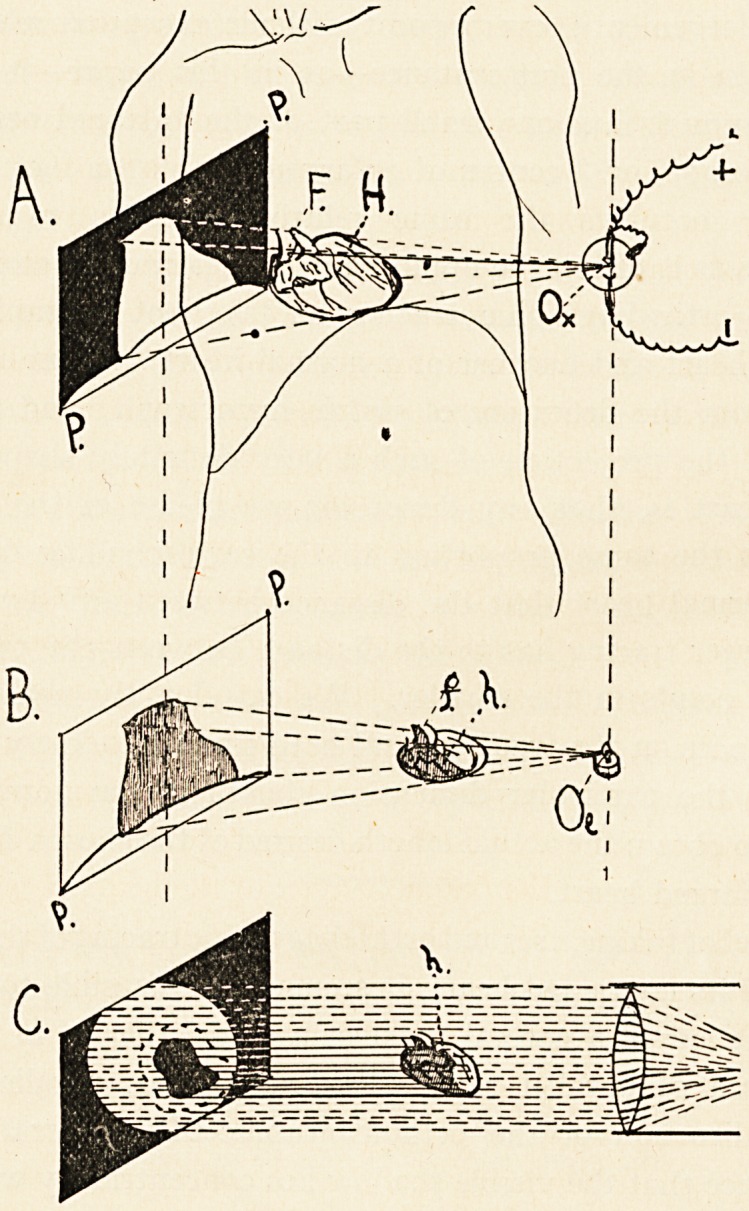# Radiographic Estimation of Simple Enlargement by Means of Visible Shadows

**Published:** 1910-09

**Authors:** William Cotton

**Affiliations:** Member of Council of the Röntgen Society.


### RADIOGRAPHIC ESTIMATION OF SIMPLE ENLARGEMENT BY MEANS OF VISIBLE SHADOWS.

BY

William Cotton, M.A., M.D., D.P.H.,

*Member of Council of the Röntgen Society.*

By simple hypertrophy of an internal organ is meant proportionate increase in size without alteration in shape, the enlarged organ being a model of the unenlarged. Whether, strictly speaking, any organ can become enlarged simply without some alteration in the way of tilting or rotation, and apart from some alteration in external shape and the interior disposition of its own parts, is questionable. It has often to be assumed that it can in the first instance, and it is so assumed for my present purpose..

In such a case as that of the heart, at any rate, every point or small part except one must occupy a different position in the enlarged organ in reference to surrounding structures than it did before enlargement ; every point without exception may do so. Here again in the first instance—as in this paper—it may be safely assumed that one small part of the enlarged organ—the " fixed point " or " centre of enlargement " of a former paper of mine[1] occupies the same relative position to adjacent structures as it did in the organ while still normal in size.

If an orthodiagraphic tracing be taken of a simple hypertrophied heart and another of a normal heart, it is at once concluded from the definition of simple hypertrophy and from the nature of the projection of such a very definitely shaped organ as the heart is, that any dimension we please of the enlarged heart has the same proportion to the corresponding dimension in the normal heart that the distance between any two points on the larger tracing has to the distance between the two corresponding points in the smaller. We can directly measure these two distances on the tracings, and need further to know only the length of the particular dimension chosen in the normal heart in order to obtain the actual length desired of the chosen dimension in the enlarged heart.

Instead of taking a second orthodiagraphic tracing of the normal chest and its contained heart, it seems to me feasible to throw a visible shadow of a normal heart or a model thereof in the open by a beam of parallel rays of ordinary light, parallel to the original direction of the orthodiagraphic X-ray, arranging for convenience that the visible shadow lies concentrically within the orthodiagraphic tracing, thus securing almost directly the same result as in the previous paragraph. By a little manipulation of

[1] " The Principle of Proportional Representation in Clinical Radiography," *Practitioner*, 1909, lxxxii, 413.

the movable testing heart in the open it would be easy to detect by imitation any tilting or twisting of the enlarged heart out of reach within the body, and failure to secure concentricity of the visible shadow with the tracing under these latter manœuvres would be diagnostic of some real change of shape in the hypertrophied heart under examination. (See diagram C, where h is the model heart).

I believe there are few of these results that cannot be as readily obtained from an ordinary radiogram of the cardiac region by the use of a visible shadow of a model heart, if only we can determine by anatomico-pathological study of, say, frozen sections the identity and actual position in regard to surrounding structures of the " fixed point " in the case of the heart, and observe certain other precautions. The determination of the position of a " fixed point " must be in many cases of enlargement of the heart unnecessary ; as where, for instance, the centre of an expansile impulse in the neighbourhood of the left nipple gives us the position of the apex of the heart, after allowance is made for the thickness of the chest wall in the direction of projection.

Geometrically it can be proved when any two similar trebly extended figures of different sizes are in perspective, the distance between any two points in the one is to the distance between the two corresponding points in the other as the distance of any point whatever in the first from the centre of perspective is to the distance of that corresponding point in the other from the centre of perspective. Algebraically, if MN be any distance in the larger, and mn the corresponding distance in the smaller, and if F and f be any other corresponding points whatever, and O be the centre of perspective, then $\frac{MN}{mn}=\frac{FO}{fO}$. Any three of these being known, the fourth can be at once found.

In Diagrams A and B I have endeavoured to show the procedure in the case of an ordinary radiogram corresponding to the procedures shown in C with an orthodiagraphic tracing.

In A, PP is the plane of delineation, H is the simply hypertrophied heart within the body, $O_x$ is the radiographic centre of emission of X-rays, F is the hypothetical " fixed point " of H (but, as I have said, the palpable apex of the heart H would often do as well). In B, PP is the plane of delineation as before in the same relative position to the centre of radiation. In this case PP is a tracing of the radiogram on the back of a print taken from the original developed negative by contact printing in the ordinary way.

$O_l$ is a small luminous flame, in the exact position as regards PP as $O_x$ was in. We may say, therefore, $O_x = O_l = O$; h is the movable model in the open, and f its " fixed point."

Now, from the nature of radiant projection, when the outline of the visible shadow of h coincides with the X-ray shadow of H, then we know that H and h, two presumably similar figures, are in the same perspective. Geometrically in accordance with the immediately previous consideration, $\frac{MN}{mn} = \frac{FO}{fO}$. Now mn and fo are directly measurable, and FO by hypothesis is calculable, and MN can then be found. If we take the palpable apex of H instead of F, then the formula becomes $\frac{MN}{mn} = \frac{AO}{aO}$, when mn and ao are directly meäsureable and AO is practically so, and A and a are the respective apices.

In the case of the radiogram, as well as in the case of the orthodiagram, the tilting of the model will give a good idea of any actual tilting or torsion of the hypertrophied organ out of reach, while likewise failure of coincidence of any part of the outline of the visible shadow with the traced radiogram on the plane of delineation, will lead to the diagnosis of some real alteration in shape.

In using the fluorescent screen instead of the developed radiogram of H, various modifications would be requisite in the procedure, the principle remaining the same. Some very subtle pitfalls would need to be shunned ; but the object of this paper is not to elaborate details, but to explain a principle of procedure. Nor is it my present object to show how the position of the "fixed point" is to be determined. Clinically it may not be found to exist ; but if it is a pathological entity, then if no better plan of determining it practically be found, I am prepared to show how it may be found in any given instance by a simple extension of the method of visible shadows suggested in this paper.

So far as the very theoretical considerations of this paper are correct under the assumptions set forth, they ought to suggest improvements in the matter of increased simplicity in practical diagnosis by means of X-rays ; and it is with this intention I submit my suggestions with regard to the solution of an important clinical problem to other workers who have larger technical opportunities and more clinical leisure than myself.

## Figures and Tables

**Figure f1:**